# The stemness of hepatocytes is maintained by high levels of lipopolysaccharide via YAP1 activation

**DOI:** 10.1186/s13287-021-02421-7

**Published:** 2021-06-10

**Authors:** Changchun Shao, Xue Yang, Yingying Jing, Xiaojuan Hou, Yihua Huang, Chen Zong, Lu Gao, Wenting Liu, Jinghua Jiang, Fei Ye, Junxia Shi, Qiudong Zhao, Rong Li, Xiaoren Zhang, Lixin Wei

**Affiliations:** 1 Tumor Immunology and Gene Therapy Center, Third Affiliated Hospital of Second Military Medical University, Shanghai, 200438 China; 2 Institute of Translational Medicine, Shanghai University, Shanghai, 200444 China; 3 Department of Pathology, The School of Basic Medical Sciences, Fujian Medical University, Fuzhou, 350108 China; 4 Affiliated Cancer Hospital and Institute of Guangzhou Medical University, Guangzhou Municipal and Guangdong Provincial Key Laboratory of Protein Modification and Degradation, State Key Laboratory of Respiratory Disease, Guangzhou, 510000 China

**Keywords:** Lipopolysaccharide, Toll-like receptor 4, Hepatocytes, Stemness maintenance, Yes-associated protein 1

## Abstract

**Background:**

The liver possesses a powerful regeneration ability, which is correlated with the stemness of hepatocytes in the portal vein (PV). However, the mechanism underlying the maintenance of hepatocyte stemness has not been elucidated. Here, we hypothesized that high levels of lipopolysaccharide from the portal vein might maintain the stemness of hepatocytes in the PV area.

**Methods:**

First, we examined the location of hepatic stem cells and the concentration of lipopolysaccharide (LPS) in the portal vein and inferior vena cava. Then, we assessed the effect of LPS on stemness maintenance in mice by using antibiotics to eliminate LPS and knocking out the LPS receptor, *TLR4*. In vitro, the effect of LPS on the stemness of hepatocytes was investigated by colony and sphere formation assays and assessment of pluripotent and stem cell marker expression. Furthermore, we studied the mechanism by which LPS regulates the stemness of hepatocytes. Finally, we ligated the portal vein branch to further verify the effect of LPS.

**Results:**

We found that a high level of LPS from the portal vein was correlated with the location of hepatic stem cells in the PV area, and elimination of LPS by antibiotics inhibited the expression of the stemness marker. LPS promoted colony and sphere formation and induced the upregulation of pluripotent and stem cell markers in AML12 cells. Furthermore, in the reprogramming medium, LPS facilitated the dedifferentiation of mature hepatocytes into hepatic progenitor-like cells, which exhibited a bipotent differentiation capacity in vivo and in vitro. Mechanistically, LPS bound TLR4 to regulate stemness of hepatocytes via the activation of YAP1 signaling, and blockade of YAP1 abolished the LPS-induced cell stemness and upregulation of pluripotent markers.

**Conclusions:**

Our study implies a correlation between LPS/TLR4/YAP1 signaling and cell stemness, and LPS was shown to be involved in stemness maintenance of hepatocytes in the PV area. LPS might be used to induce the dedifferentiation of mature hepatocytes into progenitor-like cells for repair of liver injury.

**Supplementary Information:**

The online version contains supplementary material available at 10.1186/s13287-021-02421-7.

## Background

The liver has roles in multiple processes, such as metabolism, synthesis, and biotransformation, which are critical for maintaining physiological homeostasis. Previous reports have demonstrated that native or induced liver stem cells located in the PV area after injury play a vital role in the regulation of liver homeostasis [1–4]. Thus, the PV area is thought to be the hepatic stem cell niche. However, the mechanisms responsible for the regulation of cell stemness in the PV area have not been elucidated.

The function of the liver is determined by its structure, as it comprises basic structural units and hepatic lobules [5]. The central vein is located at the center of each lobule, and the portal triad is located at the periphery. The hepatic lobule is divided into three zones along the periportal-central axis by hepatocytes with different metabolic functions: the portal vein area (PV, near the branches of the portal vein), the central vein area (CV, surrounding the central vein), and the middle area [6]. Blood flow from the periportal vein to the central vein creates gradients of oxygen, nutrients, and hormones, which might be responsible for liver zonation. Lipopolysaccharide (LPS), found in the outer membrane of gram-negative bacteria, is an important component in the portal vein, and hepatocytes are a major cell type involved in LPS uptake [7–9]. Comprehensive zone-dependent transcriptome analysis has demonstrated that the LPS response pathway shapes the characteristics of periportal hepatocytes [10]. The function of LPS uptake by hepatocytes has not yet been clearly illustrated. It is still unknown whether LPS absorption facilitates the elimination of LPS and therefore attenuates LPS-mediated injury or whether LPS internalization functions to maintain homeostasis in the portal vein area.

LPS plays an important role in liver regeneration [11–13] and in the regulation of cell stemness [14–19]. LPS pretreatment promoted hepatotrophic factor secretion and accelerated DNA synthesis for liver regeneration after partial hepatectomy (PHx) [12], and restriction of gut-derived endotoxins impaired DNA synthesis for liver regeneration after PHx [11]. Furthermore, ligation of the portal vein branches led to atrophy of the liver lobes deprived of portal blood flow and compensatory hypertrophy of the contralateral hepatic lobe [20], indicating that LPS from the portal vein might maintain liver homeostasis. Furthermore, our previous data demonstrated that LPS treatment promoted the colony and sphere formation of hepatic progenitor cells (HPCs) and inhibited HPC differentiation [21]. LPS-induced HMGB1 expression maintained the pluripotency of human embryonic stem cells and impeded their differentiation [22]. In a mouse model of acute uterine injury, LPS administration promoted the upregulation of Nanog, Sox2, and Oct4. In endothelial progenitor cells (EPCs), LPS treatment enhanced the expression of the stem cell markers AC133 and CD34, decreased the expression of differential marker eNOS, and maintained the stem cell phenotype of EPCs [14]. Furthermore, LPS was found to maintain the enteric stem cell niche by promoting the upregulation of neural stem/progenitor cell markers and the stem cell population and inhibiting their differentiation [17].

In the present study, we aimed to explore the correlation of LPS with the stemness of cells in the portal vein area, to determine whether LPS administration enhances cell stemness, and to further illustrate the mechanism underlying this phenomenon.

## Methods

### Mouse strains

Male C57BL/6 mice (6–8 weeks old) were obtained from the Shanghai Laboratory Animal Center (Shanghai, China), and TLR4 knockout (*TLR4*^*−/−*^) mice were established by CRISPR/Cas9-based genome editing at the Nanjing Xunqi Biotechnology Co., Ltd. *Fah*^*-/-*^ mice were obtained from Prof. Yiping Hu (Second Military Medical University, Shanghai, China). All animals were maintained under pathogen-free conditions, and all animal procedures were approved by the Second Military Medical University Animal Care Committee (approval number: 20175001123).

### Animal models

Mice were administered antibiotics (penicillin 1 g/L, gentamycin 1 g/L, metronidazole 1 g/L, vancomycin 500 mg/L) via their drinking water for 4 consecutive weeks to eliminate LPS from their commensal microflora [21].

For portal vein branch ligation, the left portal vein branches (PVLs) of the mice were ligated under anaesthesia, and control group mice were subjected to a sham operation. Twelve hours later, the mice were sacrificed to obtain liver samples from the sham and PVL groups.

### Cell line and culture conditions

AML12 cells were cultured in basic medium containing DMEM/F12, 10% FBS, 1% insulin-transferrin-selenium (ITS) (Sigma, UAS), 40 ng/mL dexamethasone, 1% penicillin, and 1% streptomycin according to ATCC recommendations. For short-term culture, AML12 cells were cultured in basic medium containing LPS (100 ng/mL) for the indicated amounts of time and then collected for RNA and protein analysis. For 3D culture, cells were plated on collagen-coated 12-well plates in reprogramming medium containing DMEM/F12, 1% FBS (Gibco), 10 mM nicotinamide (Sigma, UAS), 0.1 μM dexamethasone, 1% ITS, 20 ng/mL EGF (Peprotech, USA), 20 ng/mL HGF (Peprotech, USA), 10 μM Y27632 (APExBIO, USA), 1 μM A83-01 (APExBIO, USA), and 3 μM CHIR99021 (APExBIO, USA) supplemented with or without LPS (100 ng/mL) [23]. The medium was replaced every 2 days. After 2 weeks, the cells were harvested for the following assays. Mouse hepatic progenitor cells (mHPCs) were isolated and cultured as previously described [21].

### Primary hepatocyte isolation and culture

Primary hepatocytes were isolated from *WT* and *TLR4*^*-/-*^ mice by a two-step collagenase perfusion technique as previously described [23]. After preperfusion with GBSS solution through the portal vein, the liver was perfused with 50 mL of GBSS solution containing 0.05% collagenase and cut into small pieces to isolate hepatocytes. Hepatocytes were purified by a series of low-speed (1’×50 g) centrifugation steps and Percoll gradient centrifugation (50% v/v, Sigma). Purified hepatocytes were treated under 3D culture conditions.

### Differentiation assays

For hepatic differentiation, 1×10^5^ AML12 or LPS-AML12 cells were plated on collagen-coated dishes in DMEM/F12 medium containing 10% FBS, 1% penicillin/streptomycin, 20 ng/mL mouse oncostatin M (R&D Systems, MN, USA), 20 ng/mL mouse HGF (Peprotech, USA), and 10^-7^ M dexamethasone (Sigma) [24]. And the medium was changed every 2 days. After 1 week, cells were collected and used for other functional assays. For cholangiocyte differentiation, 1×10^5^ cells were seeded on collagen-coated dishes in DMEM/F12 medium supplemented with 10% FBS and 20 ng/mL HGF (Peprotech, USA) for 7 days, with fresh medium provided every 2 days [24]. After induction, cells were used for other assays.

### RNA interference

Small interfering RNAs (siRNAs) targeting the mouse TLR4 gene were designed and synthesized by RiboBio (Guangzhou, China). After overnight incubation, seeded AML12 cells were transfected with control or TLR4 siRNA (20 μM) using Lipofectamine 3000 (Invitrogen, USA) according to the manufacturer’s instructions. The pDKD-CMV-eGFP-U6-shRNA-YAP1 virus (Obio Technologies, Shanghai, China) was administered to inhibit the expression of YAP1. The target sequences are shown in Supplemental Table S1.

### Cell transplantation

AML12 cells were transfected with the pLDK-CMV-eGFP virus (Obio Technologies, Shanghai, China) for labeling with eGFP. After the administration of LPS for 2 weeks under 3D culture conditions, 1×10^6^ LPS-AML12-GFP cells were collected and transplanted into the spleens of *Fah*^*-/-*^ mice. One day before the injection, the administration of NTBC to *Fah*^*-/-*^ mice was halted to induce liver injury. After transplantation, the mice were subjected to two on-off cycles of NTBC administration (first cycle: 8 days off, 3 days on; second cycle: 7 days off, 5 days on) [23]. After 23 days, the mice were sacrificed to analyze the presence and differentiation of GFP-positive cells in the liver and liver tissue reconstitution.

### Colony formation assay

A total of 1000 AML12 cells were seeded in 6-well plates in basic medium with or without LPS for 12 days. Colonies were fixed with 4% paraformaldehyde, stained with 0.5% crystal violet, and counted.

### Sphere formation assay

A total of 1000 AML12 cells were plated in 96-well clear, flat-bottom ultralow attachment plates supplemented with or without LPS for the indicated amounts of time. The diameters of the colonies were measured under a microscope every 2 days.

### Periodic acid-Schiff (PAS) staining

After washing, the cells were fixed with 4% paraformaldehyde and stained with the periodic acid-Schiff stain kit (Servicebio, China) according to the manufacturer’s instructions.

### Endotoxin detection

Blood samples from the portal vein and inferior vena cava were harvested, and plasma was obtained by centrifugation for 10 min at 3000 rpm. Twenty-five milligrams of liver tissues from mice in the sham and PVL groups were lysed with RIPA lysis buffer to separate total proteins. The endotoxin concentrations in plasma and lysates were measured by a ToxinSensor™ Chromogenic LAL Endotoxin Assay Kit (Genscript, USA) according to the manufacturer’s protocols.

### RNA extraction and real-time PCR

The total RNA was extracted from cells using TRIzol reagent (Invitrogen, USA) according to the manufacturer’s protocols. cDNA was synthesized by a Bestar™ qPCR RT Kit (DBI, Germany), and the real-time PCR was performed using Bestar® SybrGreen qPCR mastermix (DBI, Germany) to evaluate the relative RNA expression levels of the targets. The primer sequences are listed in Supplemental Table S2.

### Western blot assay

Cells were lysed with RIPA buffer supplemented with protease and phosphatase inhibitor cocktail (Beyotime, China) to extract total proteins. After quantification, 25 μg of protein was loaded and separated with 4–12% Bis-Tris SurePAGE gels (GenScript, China) and then transferred onto PVDF membranes (Merck Millipore, Germany). After blocking with 5% BSA for 1.5 h, the membranes were incubated overnight at 4°C with anti-Sox9 (1:1000, Abcam, UK), anti-Sox2 (1:1000, Proteintech, USA), anti-Klf4 (1:1000, SAB, USA), anti-cMyc (1:1000, Novus, USA), anti-TLR4 (1:1000, Proteintech, USA), anti-YAP1 (1:1000, CST, USA), anti-CTGF (1:1000, Abcam, UK), and anti-β-actin (1:4000, Bioworld, USA) antibodies. After incubation with peroxidase-conjugated secondary antibodies (1:5000, Bioworld, USA) for 1.5 h at room temperature, the membranes were visualized using enhanced chemiluminescence detection reagents (GE Healthcare, USA).

### Immunohistochemistry (IHC) and immunofluorescence (IF) analysis

Paraffin-embedded liver samples were cut into 5-μm sections for immunohistochemistry staining according to the manufacturer’s protocol. The following antibodies were utilized: anti-YAP1 (1:200, CST, USA), anti-Sox2 (1:200, Proteintech, USA), anti-Klf4 (1:200, SAB, USA), anti-Sox9 (1:200, Abcam, UK), and anti-GS (1:2000, BD, USA). For the immunofluorescence assay, frozen sections of the liver tissues were cut into 8-μm serial sections for staining. Cells were plated on Millicell EZ SLIDE 8-well glass (Merck Millipore, Germany) and subjected to immunofluorescence staining after fixation with 4% paraformaldehyde. The slides were stained with the following antibodies: anti-Sox9 (1:200, Abcam, UK), anti-CD34 (1:100, Abbiotec, USA), anti-CK8 (1:100, Abcam, UK), anti-GS (1:1000, BD, USA), anti-TLR4 (1:200, Proteintech, USA), anti-Sox2 (1:200, Proteintech, USA), anti-Klf4 (1:200, SAB, USA), anti-cMyc (1:200, Novus, USA), anti-ALB (1:200, Abcam, UK), anti-YAP1 (1:200, CST, USA), anti-CTGF (1:200, Abcam, UK), anti-Epcam (1:200, Proteintech, USA), anti-AFP (1:200, Abcam, UK), anti-CK19 (1:200, Proteintech, USA), and anti-pan CK (1:200, Abcam, UK). The nuclei were stained with DAPI (Thermo Fisher Scientific, USA). Image-Pro Plus software (Media Cybernetics Inc., Bethesda, MD, USA) was used to measure the density of immunostaining according to previously described [25]. The mean density was calculated as follows: mean density = (IOD sum)/(area sum), where IOD represents integrated optical density.

### Statistical analysis

All experiments were performed at least three times individually. The data were analyzed using GraphPad Prism 7 (GraphPad Software Inc., CA) and are expressed as the mean ± standard deviation. The unpaired two-tailed Student’s t test was used to evaluate the statistical significance, and *P* values less than 0.05 indicated statistical significance.

## Results

### The location of hepatic stem cells is closely correlated with high concentration of LPS in the liver

**Fig. 1:**
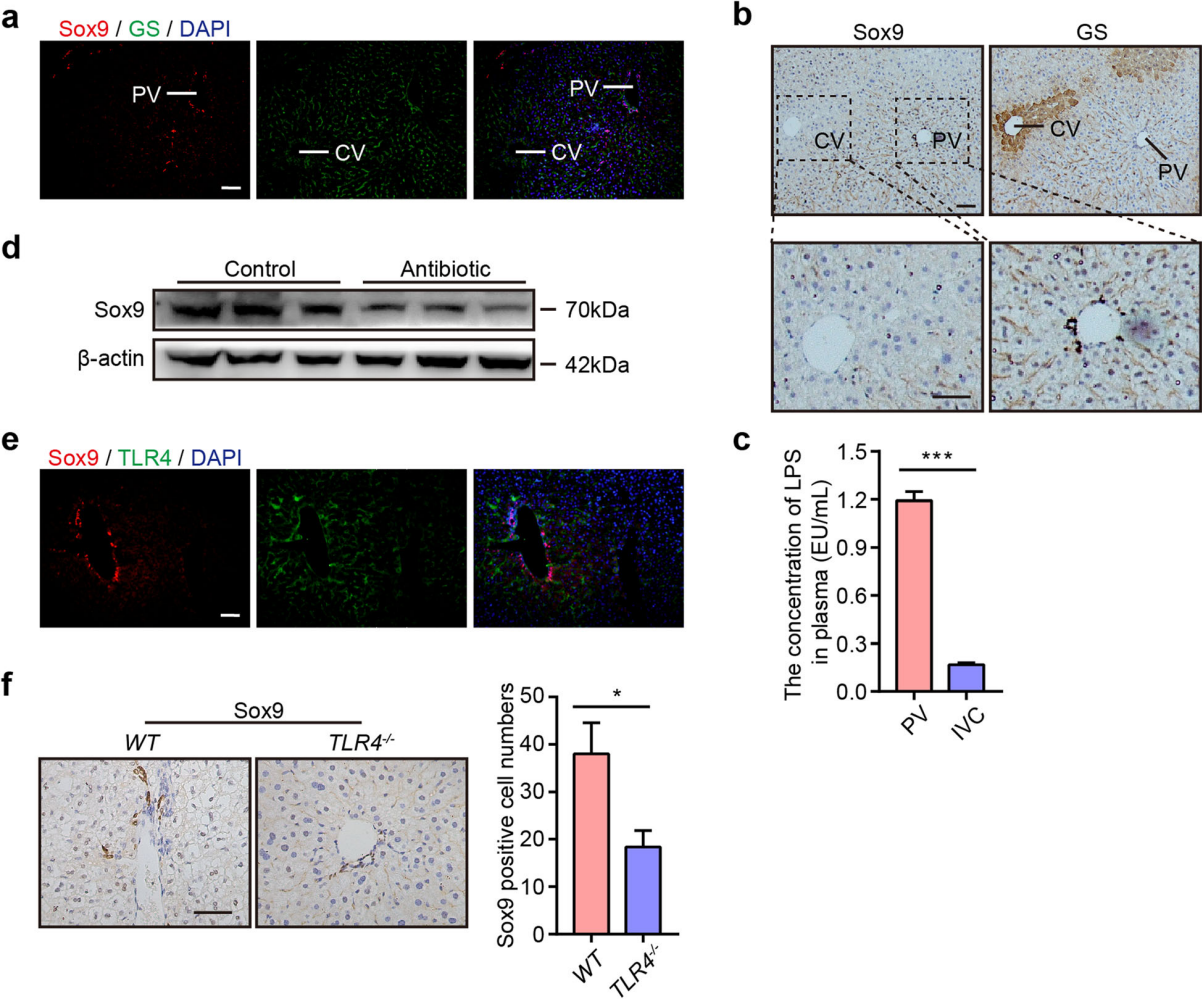
Correlation between the location of hepatic stem cells and the concentration of LPS in the liver. **a** Representative Sox9 (red) and GS (green) staining of the liver. CV, central vein; PV, portal vein. Nuclei were counterstained with DAPI (blue). **b** Representative IHC staining of serial liver sections for Sox9 and GS. **c** The concentration of LPS in the mouse portal vein (PV) and inferior vena cava (IVC). **d** Western blot assay of Sox9 in mice from the control and antibiotic groups. **e** Double staining of the liver for Sox9 and TLR4. **f** Left: Representative IHC staining of the liver for Sox9 in *WT* and *TLR4*^*-/-*^ mice; right: quantification of Sox9^+^ cell numbers. Scale bars, 100 μm. The data are expressed as the mean ± SD. *P < 0.05, ***P < 0.001

The hepatic lobule is divided into three zones, including the portal vein area (PV), central venous area (CV), and middle area, which are required for physiological liver function [26]. It has been reported that hepatic stem cells are located in the portal vein area [3, 5, 27]. First, we performed immunofluorescence (IF) staining to analyze the location of hepatic stem cells in the liver. Glutamine synthetase (GS) acts as a landmark gene for mature hepatocytes in the CV zone [26]. We found that stem cells mainly resided in the zone away from the GS^+^ area, which was supported by IF staining of Sox9, CD34, AFP, Epcam, and CK8, and the results showed that the positive cells were located in the PV area (Fig. 1a and Supplemental Figure S1a). IHC analysis of Sox9 and GS in the liver also exhibited the same result (Fig. 1b).

Our above results demonstrated that stem cells were located in the PV area. However, the regulatory mechanisms are not well understood. The PV area is fed by the portal vein, which contains abundant amounts of LPS released from enteric bacteria. Furthermore, a comprehensive zone-dependent transcriptome analysis of the liver indicated that the LPS response pathway regulated the expression of genes in the PV area [10]. To illustrate the correlation between the location of hepatic stem cells and LPS in the liver, we examined the concentration of LPS in plasma from the portal vein (PV) and inferior vena cava (IVC) (Fig. 1c). The data showed that the concentration of LPS in the PV was much higher than that in the IVC, indicating that LPS might be correlated with the distribution of hepatic stem cells in the PV area. To further confirm the correlation between LPS and hepatic stem cells, we administered antibiotics to mice to eliminate LPS. The Western blot assay revealed that the expression of Sox9 was decreased in the antibiotic group compared with the control group (Fig. 1d), which was consistent with a previous report stating that LPS regulated Sox9 expression in human periodontal ligament stem cells [16].

LPS is reported to initiate downstream signaling through its interaction with Toll-like receptor 4 (TLR4) [28]. We then performed IF staining of Sox9 and TLR4 and found that Sox9 colocalized with TLR4 in the PV area (Fig. 1e), which further confirmed the correlation between LPS and cell stemness. Next, we performed IHC and IF staining of hepatic stem cell markers Sox9, CD34, AFP, Epcam, and CK8 in *WT* and *TLR4*^*-/-*^ mice and found that their expression was much lower in *TLR4*^*-/-*^ mice than that in *WT* mice (Fig. 1f and Supplemental Figure S1b). These data implied that the location of hepatic stem cells in the portal vein area was correlated with a high level of LPS in the portal vein, which was consistent with a previous report stating that LPS played a vital role in maintaining the stem cell niche [17].

### LPS-enhanced cell stemness and promoted the dedifferentiation of hepatocytes into hepatic progenitor-like cells

**Fig. 2:**
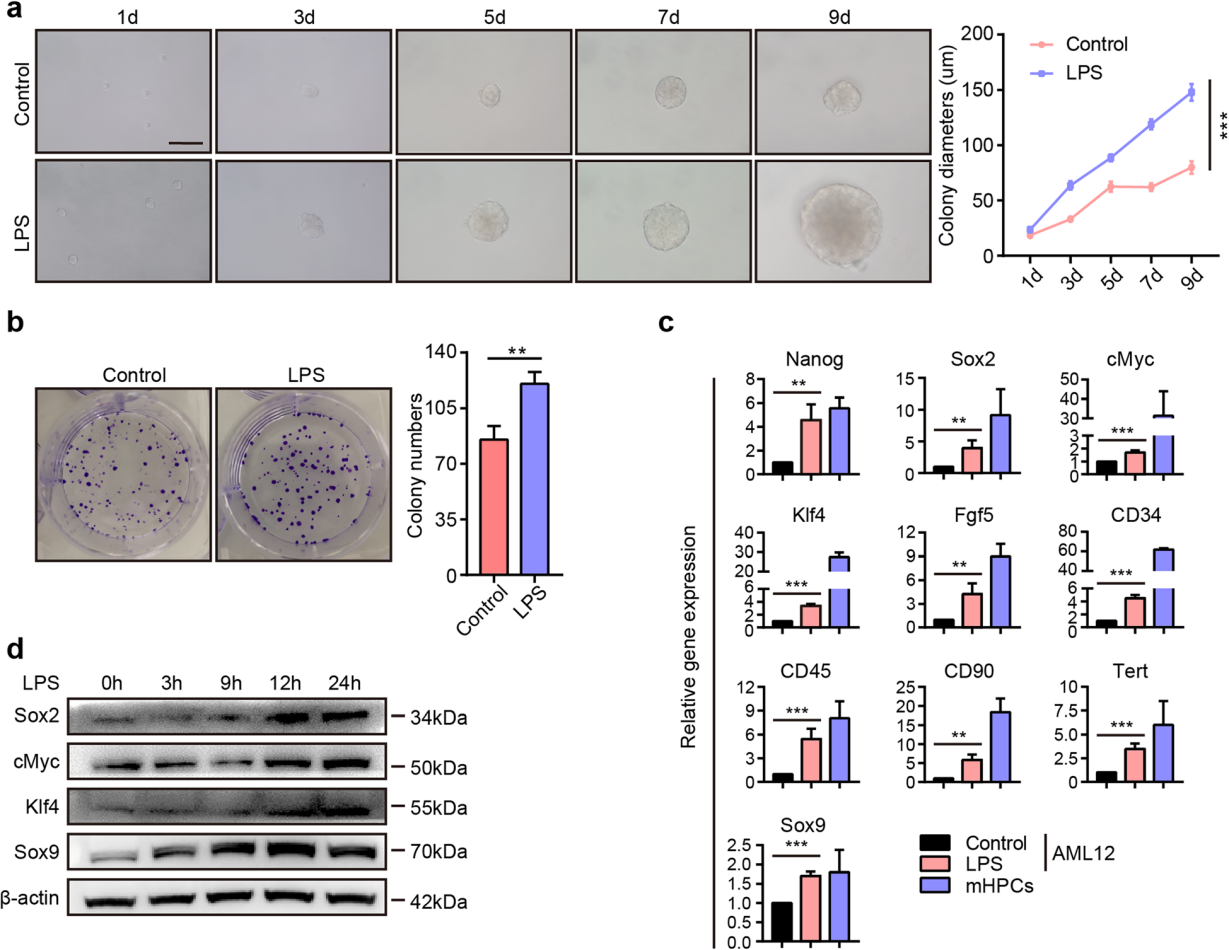
LPS promotes colony formation and pluripotent marker expression in hepatocytes. **a** Left: Representative images of AML12 cells treated with or without LPS; right: quantification of colony diameters. Scale bars, 100 μm. **b** Left: Representative images of colony formation in AML12 cells after culture with or without LPS for 12 days; right: quantification of colony numbers. **c** The relative mRNA expression of pluripotent and stem cell markers in AML12 cells cultured in basic medium in the presence or absence of LPS for 24 h. mHPCs were used as a positive control. **d** Western blot assay for Sox2, cMyc, Klf4, and Sox9 in AML12 cells cultured in basic medium containing LPS for the indicated amounts of time. h: hours; d: days. **P < 0.01, ***P < 0.001

To assess whether LPS regulated cell stemness, we performed a sphere formation assay in AML12 cells supplemented with or without LPS [9]. Strikingly, LPS promoted hepatocyte phere formation, and the colony diameters were much larger after LPS treatment (Fig. 2a). Consistently, the colony formation assay demonstrated that LPS enhanced the colony numbers in AML12 cells (Fig. 2b), suggesting that LPS was correlated with cell stemness. LPS was reported to upregulate pluripotent marker expression and induce cell stemness [29, 30]. qRT-PCR assays revealed that the pluripotent markers Nanog, Sox2, cMyc, Klf4, and Fgf5 were significantly increased, and markers associated with stem cells, such as CD34, CD45, CD90, Tert, and Sox9, were also upregulated in AML12 cells after the administration of LPS (Fig. 2c). Western blot assays revealed that LPS promoted the expression of Sox2, cMyc, Klf4, and Sox9 in AML12 cells (Fig. 2d). Furthermore, we investigated the effect of LPS on primary hepatocytes. The Western blot assay implied that after the administration of LPS, the expression of Sox2, cMyc, Klf4, and Sox9 was also upregulated (Supplemental Figure S2).

**Fig. 3:**
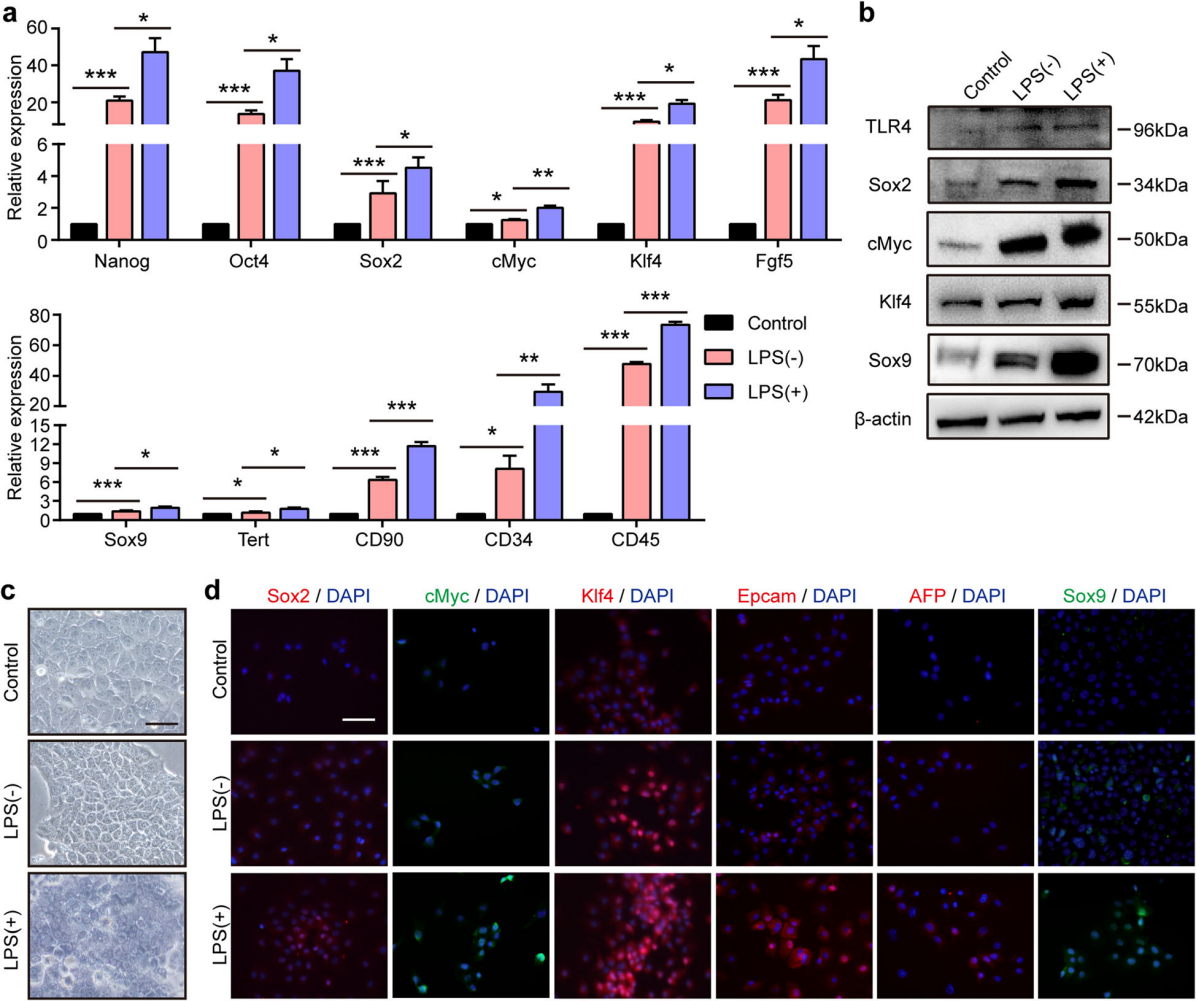
LPS supplementation enhanced hepatocytes to acquire the features of hepatic progenitor-like cells in the reprogramming medium. **a** The relative mRNA expression of pluripotent and stem cell markers in AML12 cells cultured in basic medium and in AML12 cells cultured in reprogramming medium with or without LPS for 2 weeks. Control: AML12 cells were cultured in basic medium; LPS (−): AML12 cells were cultured in reprogramming medium without LPS; LPS (+): AML12 cells were cultured in reprogramming medium containing LPS. **b** Western blot assay of TLR4, Sox2, cMyc, Klf4, and Sox9 in AML12 cells in the control, LPS (−), and LPS (+) groups. **c**, **d** Representative phase-contrast (**c**) and immunofluorescence images of staining for pluripotent and hepatic progenitor cell markers (**d**) in AML12 cells in the control, LPS (−), and LPS (+) groups. Scale bars, 50 μm. *P < 0.05, **P < 0.01, ***P < 0.001

Recent lineage-tracing studies have shown that mature hepatocytes can be converted into hepatic progenitor-like cells under the condition of chronic periportal liver injury [4]. An in vitro assay demonstrated that a cocktail of small molecules could induce the conversion of mature hepatocytes into bipotent stem cells [24]. Our above data demonstrated that LPS promoted the expression of pluripotent genes and colony formation. Whether LPS promotes the conversion of mature hepatocytes into progenitor-like cells has not been elucidated. To address this question, we cultured AML12 cells with reprogramming medium with or without LPS. qRT-PCR assays demonstrated that the pluripotent genes Nanog, Oct4, Sox2, cMyc, Klf4, and Fgf5 were enriched in AML12 cells after culture in the reprogramming medium, and the stem cell markers Sox9, Tert, CD90, CD34, and CD45 were also increased (Fig. 3a). Additionally, the expression of pluripotent and stem cell markers was further induced by supplementation of the reprogramming medium with LPS. Consistently, the Western blot assay showed that AML12 cells in the reprogramming medium containing LPS displayed much higher levels of the pluripotent genes Sox2, cMyc, and Klf4 and the stem cell marker Sox9 (Fig. 3b). TLR4 expression was much higher in AML12 cells cultured in reprogramming medium containing LPS. AML12 cells exposed to reprogramming medium containing LPS exhibited a typical phenotype of progenitor-like cells with a high nucleus/cytoplasm ratio (Fig. 3c). IF staining demonstrated that after the administration of LPS, the expression levels of the pluripotent genes Sox2, cMyc, and Klf4 and the hepatic progenitor cell genes Epcam, AFP, and Sox9 in AML12 cells were much higher (Fig. 3d and Supplemental Figure S3). Chemically induced hepatic progenitors exhibited the ability to differentiate into both hepatocytes and cholangiocytes [24], and we further investigated whether these LPS-induced AML12 cells exhibited a bipotent differentiation ability.

### Bipotentiality of LPS-induced hepatocytes to convert into hepatocytes and cholangiocytes in vitro and in vivo

**Fig. 4:**
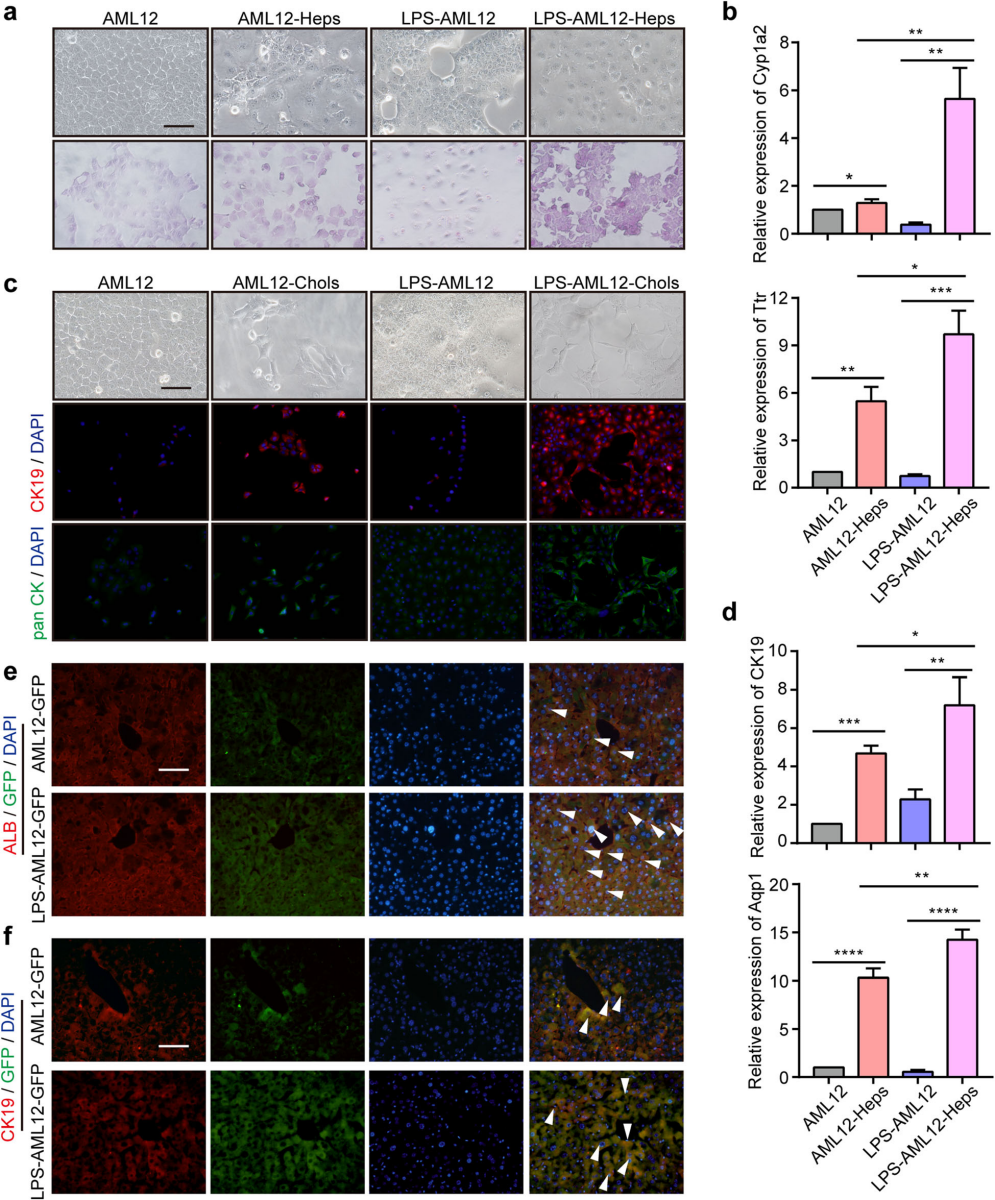
LPS-induced hepatocytes exhibit bipotent differentiation potential in vitro and in vivo. **a** Upper: Representative phase-contrast images of AML12 and LPS-AML12 cells with (right) or without (left) hepatic induction. Bottom: Representative images of PAS staining. Scale bars, 50 μm. **b** The relative expression of the mature hepatic marker Cyp1a2 and Ttr in AML12 cells, AML12-Heps, LPS-AML12 cells, and LPS-AML12-Heps as determined by qRT-PCR. **c** Representative phase-contrast images and IF staining of CK19 and pan CK in AML12 cells and LPS-AML12 cells with (right) and without (left) biliary induction. Scale bars, 50 μm. **d** qRT-PCR analysis of the cholangiocyte marker CK19 and Aqp1 in AML12 cells, AML12-Chols, LPS-AML12, and LPS-AML12-Chols. **e** IF staining of the mature hepatocyte markers ALB (red) . The arrowheads denote AML12-GFP cells and LPS-AML12-GFP cells with ALB staining. Scale bars, 50 μm. **f** IF staining of live chimaeric *Fah*^*-/-*^ mice for the cholangiocyte markers CK19 (red). The arrowheads denote AML12-GFP cells and LPS-AML12-GFP cells with CK19 staining. LPS-AML12: AML12 cells were cultured in reprogramming medium in the presence of LPS for 2 weeks. Scale bars, 50 μm. *P < 0.05, **P < 0.01, ***P < 0.001

To evaluate the differentiation potential of LPS-induced hepatocytes (LPS-AML12), we performed lineage-specific differentiation assays [31]. After hepatic induction, LPS-AML12 cells exhibited a typical mature hepatocyte morphology (Fig. 4a, upper). PAS staining was performed to evaluate the glycogen storage capacity of mature hepatocytes. We found that the glycogen storage capacity was enhanced in LPS-AML12 cells after hepatic induction and was much higher than that in AML12-Heps (Fig. 4a, bottom). qRT-PCR assays indicated that LPS-AML12 cells exhibited comparable induction of the mature hepatocyte marker Cyp1a2, Ttr, Alb, and Hnf4α after hepatic induction (Fig. 4b and Supplemental Figure S4a). Furthermore, we found that LPS-AML12 cells could form a bile duct-like structure after cholangiocyte induction (Fig. 4c). Consistently, IF staining of the cholangiocyte markers CK19 and pan CK revealed that their expression was comparably increased in LPS-AML12 cells after induction and was much higher than that in AML12 cells after induction (Fig. 4c). qRT-PCR analysis of CK19, Aqp1, and Aqp9 also confirmed these results, suggesting that LPS-AML12 cells could be induced to differentiate into bile duct-like cells (Fig. 4d and Supplemental Figure S4b).

To further evaluate the differentiation potential of LPS-AML12 cells in vivo, we transplanted AML12 and LPS-AML12 cells into the spleens of *Fah*^*-/-*^ mice. First, we transfected AML12 cells with the pLDK-CMV-eGFP virus for labeling with eGFP. After induction with reprogramming medium containing LPS, LPS-AML12-GFP cells were injected into the spleens of *Fah*^*-/-*^ mice. Twenty-three days posttransplantation, the mice were sacrificed to evaluate the differentiation potential of AML12-GFP and LPS-AML12-GFP cells. IF staining indicated that the LPS-AML12-GFP cells exhibited extensive engraftment, in contrast to the modest engraftment after transplantation of AML12-GFP cells (Supplemental Figure S4c). IF staining of the mature hepatocyte markers ALB and GS demonstrated that GFP-positive cells exhibited ALB or GS staining (Fig. 4e and Supplemental Figure S4d), suggesting that LPS-AML12 cells possessed the ability to differentiate into hepatocytes. Furthermore, GFP-positive cells were partially colocalized with the cholangiocyte markers CK19 and pan CK, and the percentages of CK19^+^GFP^+^ and pan CK^+^GFP^+^ cells after transplantation of LPS-AML12-GFP cells were much greater than those after transplantation of AML12-GFP (Fig. 4f and Supplemental Figure S4e). Our data indicated that LPS promoted AML12 cells to convert into hepatic progenitor-like cells, which exhibited bipotent differentiation potential in vitro and in vivo.

### LPS-induced hepatocyte reprogramming is dependent on the TLR4 pathway

**Fig. 5:**
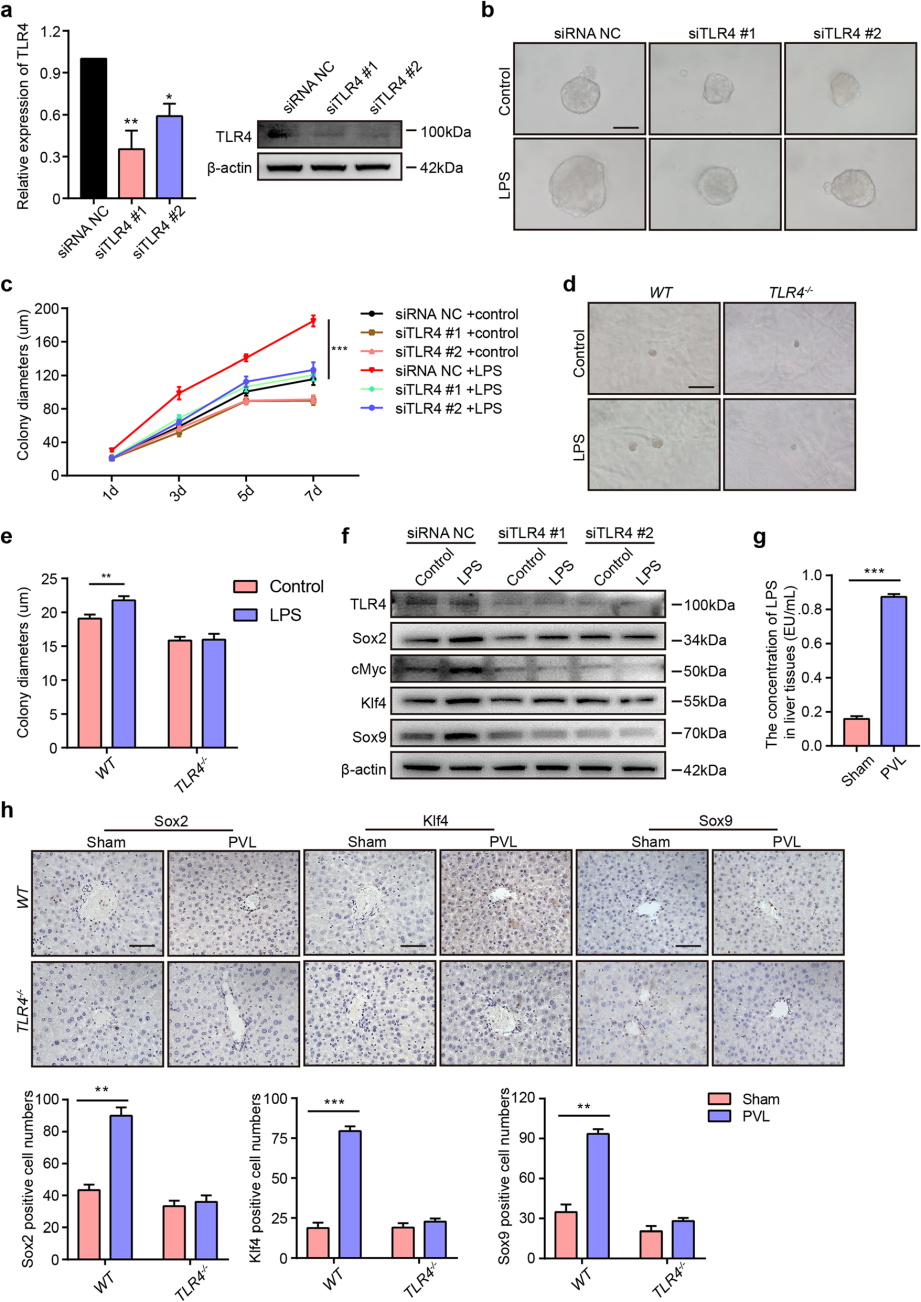
LPS promotes colony formation and pluripotent marker expression via the TLR4 pathway. **a** The relative expression of TLR4 at the RNA and protein levels in AML12 cells after transfection with siRNA NC, siTLR4 #1, and siTLR4 #2. **b**, **c** After transfection with siRNA NC or siTLR4 for 24 h, AML12 cells were plated and cultured with or without LPS for 7 days. Representative images of colonies (**b**) and the quantification of colony diameters (**c**). **d** Representative images of primary hepatocytes from *WT* and *TLR4*^*-/-*^ mice cultured with or without LPS for 2 weeks. **e** Quantification of the colony diameters of primary hepatocytes from *WT* or *TLR4*^*-/-*^ mice. **f** After transfection with siRNA NC or siTLR4 for 24 h, AML12 cells were cultured with LPS for 24 h and lysed to analyze the expression of TLR4, Sox2, cMyc, Klf4, and Sox9 by Western blot. **g** After ligation of the left portal vein branch, the right liver lobes were obtained from the sham and PVL groups. The concentrations of LPS in the right liver lobes of *WT* mice from the sham and PVL groups. **h** Upper: IHC staining of Sox2, Klf4, and Sox9 in the right liver lobes of *WT* and *TLR4*^*-/-*^ mice in the sham and PVL groups. Bottom: Quantification of the Sox2-, Klf4-, and Sox9-positive cell numbers. Scale bars, 50 μm. d: days. *P < 0.05, **P < 0.01, ***P < 0.001

It was reported that murine hepatocytes expressed TLRs and responded to LPS through a TLR4 response pathway [32]. To investigate whether LPS combined with TLR4 regulates sphere formation and pluripotent marker expression in vitro, we administered TLR4 siRNA to AML12 cells. After transfection with TLR4 siRNA, the expression of TLR4 at the RNA and protein levels was downregulated (Fig. 5a). The sphere formation assay showed that LPS treatment significantly promoted the increase in colony size in the control group, but LPS failed to increase the colony size in the siTLR4 group (Fig. 5b, c). To further strengthen the evidence regarding this phenomenon, we performed a sphere formation assay of primary hepatocytes from *WT* and *TLR4*^*-/-*^ mice. Consistently, the results demonstrated that LPS enhanced the sphere formation of primary hepatocytes in the presence of TLR4 but failed to promote the sphere formation of primary hepatocytes in the absence of TLR4 (Fig. 5d, e). Western blot assays demonstrated that LPS promoted the expression of the pluripotent markers Sox2, cMyc, and Klf4 and the stem cell marker Sox9 in the control group, but LPS failed to induce an increase in Sox2, cMyc, Klf4, and Sox9 expression in AML12 cells after silencing TLR4 (Fig. 5f). Our results showed that TLR4 signaling was required for LPS-induced sphere formation and pluripotent marker expression.

The liver possesses a dual vascular system that supplies blood throughout the organ, including to the portal vein (70–80%) and hepatic artery (20–30%). Portal blood comes from the gastrointestinal tract and contains abundant amounts of LPS. To further illustrate the function of LPS from the portal vein in vivo, we ligated the left portal vein branch and analyzed the expression of genes in the right hepatic lobes from *WT* and *TLR4*^*-/-*^ mice. After ligation of the left portal vein branch (PVL), the concentration of LPS in the right hepatic lobes was much higher in the PVL group than in the sham group (Fig. 5g). IHC staining of Sox2, Klf4, and Sox9 demonstrated that the number of Sox2^+^, Klf4^+^, and Sox9^+^ cells was increased in the PVL group of *WT* mice, but there was no significance in the numbers in *TLR4*^*-/-*^ mice, and the positive cells were all located in the PV area (Fig. 5h), suggesting that LPS in the portal vein regulates the expression of pluripotent markers via the TLR4 pathway.

### YAP1 acts as a downstream target of LPS/TLR4 signaling to regulate hepatocyte stemness

**Fig. 6:**
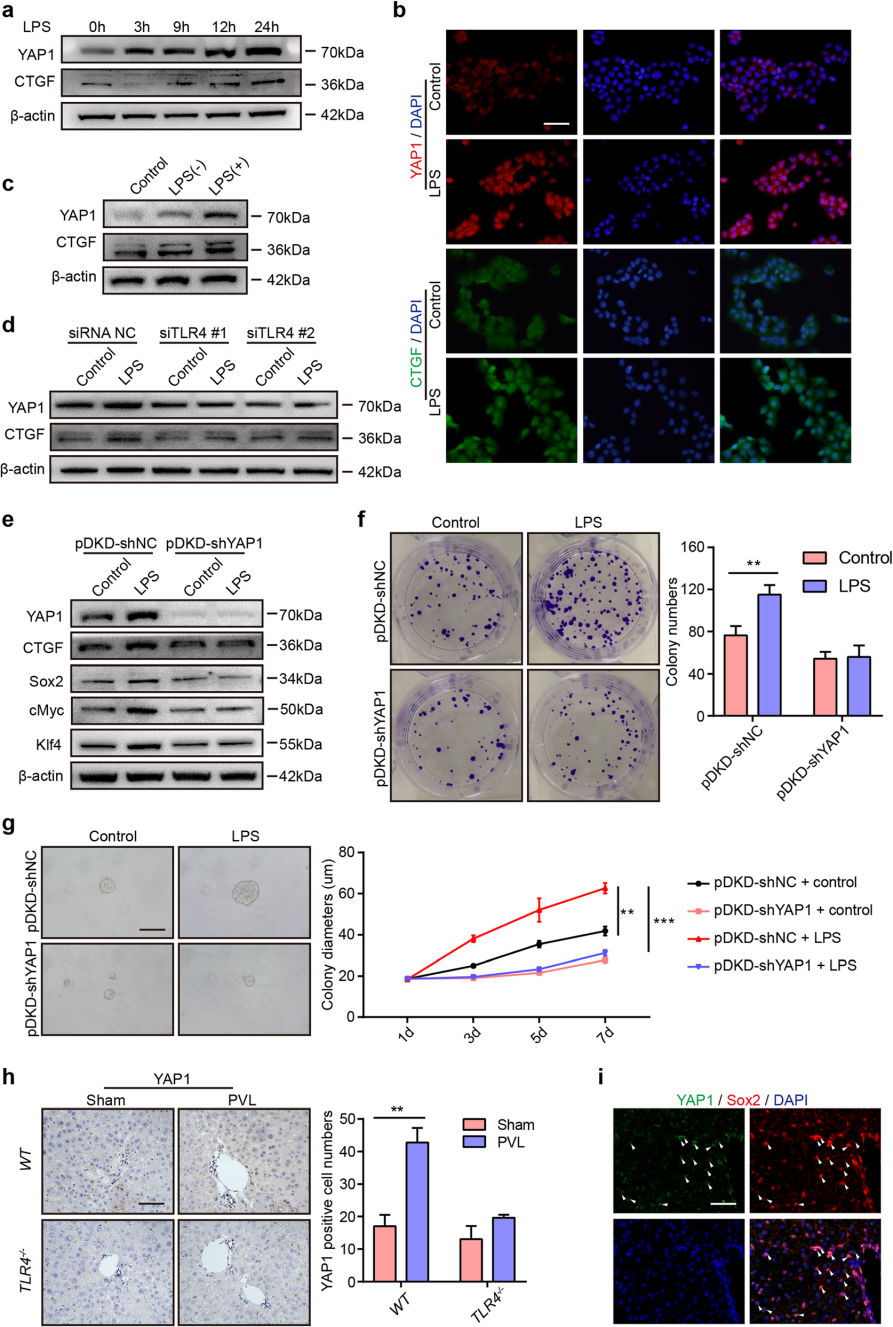
YAP1 plays an essential role in cell stemness driven by LPS/TLR4 signaling. **a** Western blot analysis of YAP1 and CTGF expression in AML12 cells cultured in basic medium containing LPS for the indicated amounts of time. **b** IF staining of YAP1 and CTGF in AML12 cells after the administration of LPS for 24 h. **c** Western blot assay of YAP1 and CTGF in AML12 cells in the different groups. Control: AML12 cells cultured in basic medium; LPS (−): AML12 cells cultured in reprogramming medium without LPS; LPS (+): AML12 cells cultured in reprogramming medium containing LPS. **d** Western blot assay of YAP1 and CTGF in AML12 cells cultured in basic medium with or without LPS for 24 h after transfection with siRNA NC or siTLR4. **e** After transfection with the pDKD-shNC or PDKD-shYAP1 virus for 48 h, AML12 cells were cultured in basic medium with or without LPS for 24 h and then lysed to analyze the protein expression of YAP1, CTGF, Sox2, cMyc, and Klf4. **f** Left: Representative images of the colony formation of AML12 cells transfected with the pDKD-shNC or pDKD-shYAP1 virus after culture with or without LPS for 12 days; right: quantification of the colony numbers. **g** After transfection with the pDKD-shNC or pDKD-shYAP1 virus for 48 h, AML12 cells were cultured with or without LPS for 7 days to evaluate sphere formation. Left: Representative images of sphere formation; right: quantification of the colony diameters. **h** Left: IHC staining of YAP1 in the right liver lobes *WT* and *TLR4*^*-/-*^ mice from the sham and PVL groups. Bottom: Quantification of the YAP1-positive cell numbers. **i** IF staining of YAP1 and Sox2 in the right liver lobes of *WT* mice from the PVL group. PVL, ligation of the left portal vein branch. Scale bars, 50 μm. h: hours; d: days. **P < 0.01, ***P < 0.001

Hippo/YAP1 pathway activity is essential for the maintenance of the differentiated hepatocyte state, and activation of YAP1 promotes committed cells to dedifferentiate back to a progenitor and stem cell state [33, 34], whether YAP1 participates in LPS-induced stemness in hepatocytes is not clearly illustrated. To evaluate the function of YAP1 in the process, we performed a Western blot assay to examine the expression of YAP1 in AML12 cells after the administration of LPS. We found that YAP1 expression was highly enriched, and CTGF, the most highly characterized YAP1 target gene, was also increased in AML12 cells after culture with LPS (Fig. 6a). IF staining for YAP1 and CTGF indicated that LPS promoted YAP1 expression and increased the nuclear localization of YAP1 in AML12 cells, and CTGF expression was also upregulated after LPS administration (Fig. 6b). Furthermore, YAP1 and CTGF expression was much higher in AML12 cells cultured in reprogramming medium containing LPS (Fig. 6c), suggesting that LPS promoted YAP1 activation. Additionally, we examined the expression of YAP1 after silencing TLR4 in AML12 cells. The data showed that LPS failed to activate YAP1 and CTGF after TLR4 was silenced, demonstrating that LPS induced the activation of YAP1 signaling, which was dependent on the TLR4 pathway (Fig. 6d).

To further illustrate the role of YAP1 in the process, we knocked down the expression of YAP1 with adenovirus. Western blot assays demonstrated that YAP1 was downregulated after administration of the pDKD-shYAP1 virus, and LPS failed to induce YAP1 activation in AML12 cells after transfection with the pDKD-shYAP1 virus (Fig. 6e). Consistently, LPS promoted the upregulation of CTGF, Sox2, cMyc, and Klf4 in AML12 cells transfected with the pDKD-shNC virus, but LPS failed to induce the upregulation of these genes in AML12 cells after transfection with the pDKD-shYAP1 virus, indicating that LPS might promote cell stemness via YAP1 signaling. To confirm this phenomenon, we performed colony and sphere formation assays. The results showed that LPS promoted colony formation (Fig. 6f) and sphere formation (Fig. 6g) in pDKD-shNC-treated AML12 cells but not in pDKD-shYAP1-treated AML12 cells. Our results demonstrated that LPS regulated colony formation and cell stemness via YAP1 activation. Furthermore, we examined the expression of YAP1 in the model of the left portal vein branch ligation in *WT* and *TLR4*^*-/-*^ mice. IHC staining of YAP1 showed that the number of YAP1^+^ cells was increased in the PVL group of *WT* mice but not in *TLR4*^*-/-*^ mice, indicating that LPS could facilitate the expression of YAP1 (Fig. 6h). YAP1 and Sox2 double staining of liver lobes in the PVL group from *WT* mice showed that YAP1 and Sox2 were colocalized (Fig. 6i), suggesting that YAP1 collaborates with Sox2 to help regulate cell stemness induced by LPS.

### Correlation among LPS, YAP1, and Sox9 expression *in the liver*

Our above data demonstrated that a high concentration of LPS was correlated with the location of hepatic stem cells in the PV area, and LPS promoted the expression of pluripotent markers and cell stemness via the activation of YAP1 signaling. Whether LPS is essential for the maintenance of hepatocyte stemness in the PV area has not been fully elucidated. To answer this question, we ligated the left portal vein branch in the left liver lobes and analyzed the expression of YAP1 and Sox9; the expression of YAP1 and Sox9 in the right liver lobes served as controls.

**Fig. 7:**
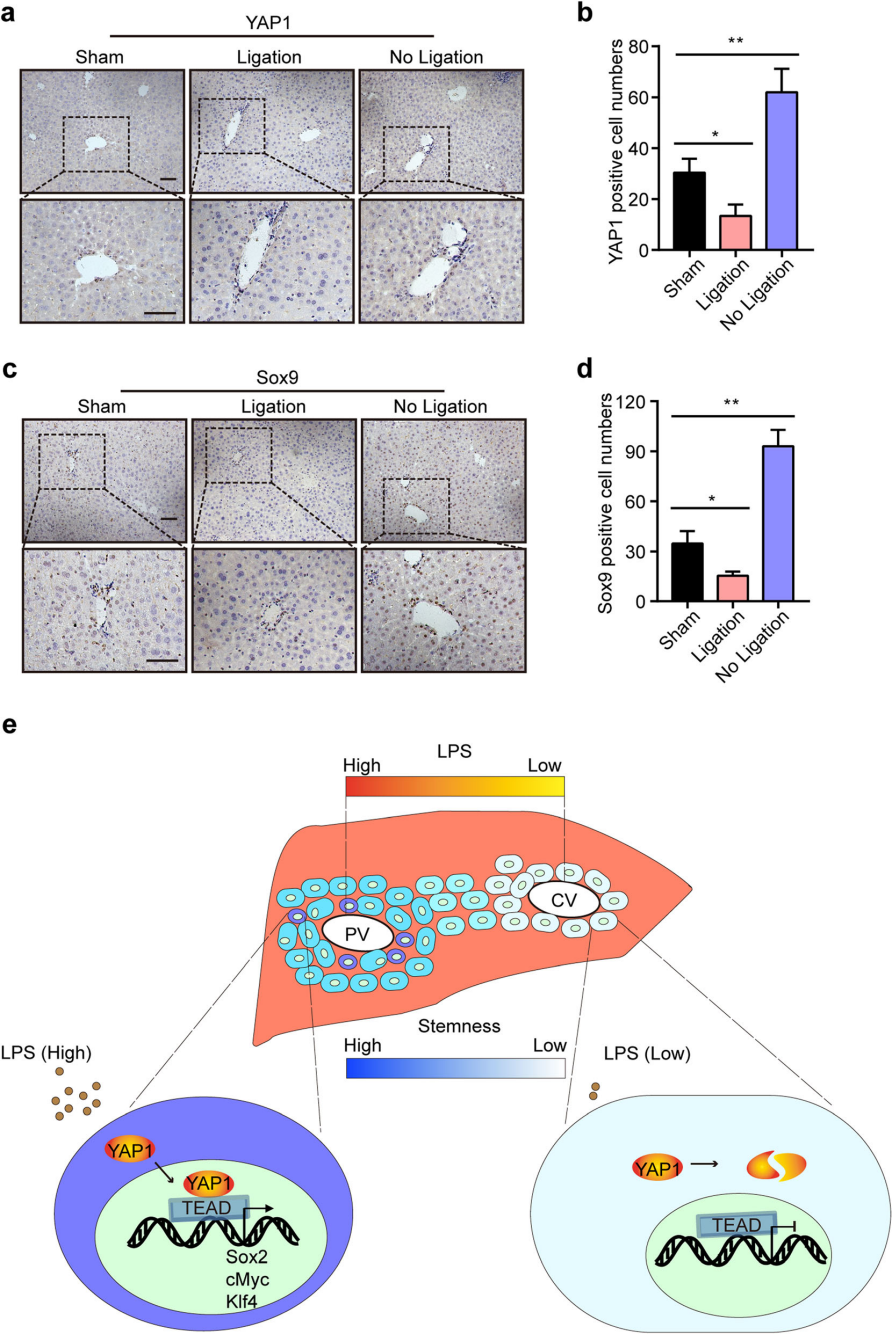
Correlation among LPS, YAP1, and Sox9^+^ stem cells *in vivo*. **a**, **b** Mice were administered a sham surgery or subjected to ligation of the left portal vein branch for 12 h and then sacrificed to harvest their liver lobes. IHC staining of YAP1 in the different liver lobes of *WT* mice in the sham and PVL groups (**a**). Quantification of the YAP1-positive cell numbers (**b**). Ligation: the left liver lobes with ligation of the portal vein from the PVL group; no ligation: the right liver lobes with no ligation of the portal vein from the PVL group. **c**, **d** IHC staining of Sox9 in the different liver lobes of mice in the sham and PVL groups (**c**) and the quantification of the Sox9-positive cell numbers (**d**). **e** Schematic representation of the LPS/YAP1 axis-mediated maintenance of hepatocyte stemness in the PV area. Scale bars, 50 μm. *P < 0.05, **P < 0.01

After ligation of the left portal vein branch, the concentration of LPS in the ligated liver lobes was much lower than that in the sham group (Supplemental Figure S5). IHC staining of YAP1 and Sox9 demonstrated that the number of YAP1^+^ and Sox9^+^ cells was decreased in the ligated liver lobes but increased in the liver lobes with no ligation, and the positive cells were all located in the PV area (Fig. 7a–d), suggesting that LPS in the portal vein played an essential role in the maintenance of hepatocyte stemness in the PV area (Fig. 7e).

## Discussion

Liver stem cells play an important role in maintaining liver homeostasis, as they can differentiate into hepatocytes and cholangiocytes and repopulate the injured liver to restore liver mass and function [35, 36]. Growing evidence supports that LPS involves in liver regeneration and stem cell phenotype maintenance [11, 12, 15, 17]. However, the effect of LPS on maintaining hepatocyte stemness in the PV area has not been elucidated. Our results revealed that LPS in the portal vein might participated in the stemness maintenance of hepatocyte in the PV area and that LPS administration enhanced stemness and promoted the expression of pluripotent and hepatic progenitor cell markers in hepatocytes. LPS treatment facilitated hepatocytes to acquire the characteristics of hepatic progenitor cells via YAP1 activation under reprogramming conditions, and LPS-induced hepatic progenitor-like cells exhibited a bipotent differentiation ability in vitro and in vivo.

Previous reports have indicated that intrahepatic stem cell niches are the canals of Hering in the PV area, and there are at least 8 maturational lineage stages ranging from the stem cells in the PV area through the midacinar region to the most mature cells and apoptotic cells in the CV area [37]. However, the mechanism underlying the location of stem cells is not clearly illustrated. Here, the concentration gradient of LPS from the PV area to the CV area in the lobule was correlated with the distribution of hepatic stem cells. Hepatic stem cells resided in the PV area, where the concentration of LPS in the portal vein was much higher than that in the inferior vena cava draining from the central vein. After the administration of antibiotics, the expression of stem cell marker in the liver was downregulated, suggesting that LPS might regulate cell stemness. Consistently, LPS has been reported to participate in maintaining the stem cell phenotype in enteric neural stem/progenitor cells, dental pulp stem cells, and endothelial progenitor cells [14, 15, 17].

LPS administration was found to upregulate the expression of the pluripotent markers Nanog, Sox2, and Oct4 in a mouse model of acute uterine injury [29]. It was reported that these transcription factors could induce the conversion of primary hepatocytes into pluripotent stem cells [38, 39]. Our study revealed that LPS treatment promoted colony and sphere formation and induced the upregulation of the pluripotent markers Sox2, Klf4, and cMyc and the stemness genes Sox9 and Epcam in hepatocytes, suggesting that LPS can enhance the stemness of hepatocytes. Small molecules could directly convert terminally mature hepatocytes into bipotent hepatic progenitor cells [24, 31]. In our study, we utilized a reprogramming culture system supplemented with LPS to illustrate the function of LPS in this process. Our results suggested that LPS further promoted the expression of pluripotent and stem cell markers and facilitated the mature hepatocytes to acquire the features of hepatic progenitor cells, which harbored the capacity to differentiate into hepatocytes and BECs in vitro and in vivo, as determined by cell morphology and molecular marker assessments. Additionally, LPS-AML12 cells repopulated the injured livers of *Fah*^*-/-*^ mice after the withdrawal of NTBC, implying their potential capability for repopulating the injured liver.

The YAP1 signaling pathway has an important role in the regulation of organ size and cell fate [40, 41]. It was reported that overexpression of YAP1/TAZ induced the conversion of primary differentiated mammary gland, neuronal, and pancreatic exocrine cells into a stem/progenitor cell state with high proliferation potential [34]. Additionally, YAP1 activation promoted the differentiation of adult hepatocytes into progenitor cells with self-renewal and engraftment capacity [33], and YAP1 inactivation upregulated liver-enriched gene expression and facilitated the functional differentiation of induced hepatocyte-like cells [42]. In the current study, LPS administration enhanced the expression of YAP1 and the downstream gene CTGF and induced the translocation of YAP1 into the nucleus. Moreover, LPS-induced cell stemness was abolished after silencing YAP1 via pDKD-shYAP1, suggesting that YAP1 might participate in LPS-induced cell stemness. Additionally, we found that LPS failed to upregulate YAP1 expression after knocking down TLR4 using small interfering RNA, implying that LPS activated YAP1 expression through TLR4 signaling. However, the mechanism by which LPS induces the activation of YAP1 needs to be further investigated.

The gene expression profile in the portal vein area suggests that the LPS signaling pathway regulates the characteristics of periportal cells [10]. To further examine the effect of LPS on the expression of pluripotent markers in vivo, we performed portal vein ligation in *WT* and *TLR4*^*-/-*^ mice. Ligation of the portal vein branches led to the flow of portal blood into the contralateral hepatic lobes, and our results revealed that the upregulation of pluripotent markers in the contralateral hepatic lobes of *WT* mice but not in those of *TLR4*^*-/-*^ mice. The positive cells were located in the PV area, suggesting that LPS promoted the expression of pluripotent markers via TLR4 in vivo, which was consistent with the above data that LPS failed to promote sphere formation and the expression of pluripotent markers after silencing TLR4 in hepatocytes in vitro.

## Conclusions

To conclude, our study illustrated the important role of LPS in the regulation of hepatocyte stemness. High levels of LPS from the portal vein might maintain liver homeostasis and regulate cell stemness in the PV area through TLR4 signaling. YAP1 was found to be involved in the LPS/TLR4-induced stemness of hepatocytes. Furthermore, LPS facilitates the conversion of mature hepatocytes into progenitor-like cells with bipotent differentiation potential and thus might have potential applications for repair of liver injury.

## Supplementary Information

**Additional file 1: Supplemental Figure S1.** The expression of the hepatic stem cell markers in *WT* and *TLR4*^*-/-*^ mice. a IF staining of the hepatic stem cell markers CD34 (red), AFP (red), Epcam (red), and CK8 (red) in the liver. Nuclei were counterstained with DAPI (blue). Scale bars, 100 μm. b IF staining of the hepatic stem cell markers CD34, AFP, Epcam, and CK8 in the liver of *WT* and *TLR4*^*-/-*^ mice. Mean density was used to evaluate the expression of these markers. Mean density was calculated as follows: Mean density = (IOD Sum) / (Area Sum), where IOD represents integrated optical density. Scale bars, 50 μm.

**Additional file 2: Supplemental Figure S2.** Western blot assay of Sox2, cMyc, Klf4, and Sox9 in primary hepatocytes cultured in basic medium containing LPS for the indicated amounts of time.

**Additional file 3: Supplemental Figure S3.** The expression of Sox2, cMyc, Klf4, Epcam, AFP, and Sox9 was measured by mean density.

**Additional file 4: Supplemental Figure S4.** The relative expression of hepatic or biliary markers in LPS-AML12 cells after hepatic or biliary induction. a The relative expression of the mature hepatic marker Alb and Hnf4α in AML12 cells, AML12-Heps, LPS-AML12 cells, and LPS-AML12-Heps as determined by qRT-PCR. b qRT-PCR analysis of the cholangiocyte marker Aqp9 in AML12 cells, AML12-Chols, LPS-AML12, and LPS-AML12-Chols. c Colonization of GFP-tagged AML12 or LPS-AML12 cells in *Fah*^-/-^ mice 23 days after transplantation. Scale bars, 500 µm. d IF staining of the mature hepatocyte markers GS (red). The arrowheads denote AML12-GFP cells and LPS-AML12-GFP cells with GS staining. Scale bars, 50 µm. e IF staining of live chimaeric *Fah*^-/-^ mice for the cholangiocyte markers pan CK (red). The arrowheads denote AML12-GFP cells and LPS-AML12-GFP cells with pan CK staining. LPS-AML12: AML12 cells were cultured in reprogramming medium in the presence of LPS for 2 weeks. Scale bars, 50 µm. *P < 0.05, **P < 0.01, ***P < 0.001, ****P < 0.0001.

**Additional file 5: Supplemental Figure S5.** The concentrations of LPS in the different liver lobes from *WT* mice. **P < 0.01, ***P < 0.001.

**Additional file 6: Supplemental Table S1.** siRNA target sequences.

**Additional file 7: Supplemental Table S2.** Real-time PCR primers.

## Data Availability

The datasets generated for this study are available on request to the corresponding author.

## Abbreviations

LPS: Lipopolysaccharide
YAP1: Yes-associated protein 1
TLR4: Toll-like receptor 4
mHPCs: Mouse hepatic progenitor cells
PV: Portal vein
CV: Central vein
PHx: Partial hepatectomy
EPCs: Endothelial progenitor cells
PVL: Ligation of the portal vein
PAS: Periodic acid-Schiff (PAS) staining
IHC: Immunohistochemistry assay
IF: Immunofluorescence assay
GS: Glutamine synthetase
