# Supplementary figures and images for: The stemness of hepatocytes is maintained by high levels of lipopolysaccharide via YAP1 activation
Source: Stem Cell Res Ther. 2021 Jun 10;12:342. doi: 10.1186/s13287-021-02421-7 (PMC8193885; doi:10.1186/s13287-021-02421-7)

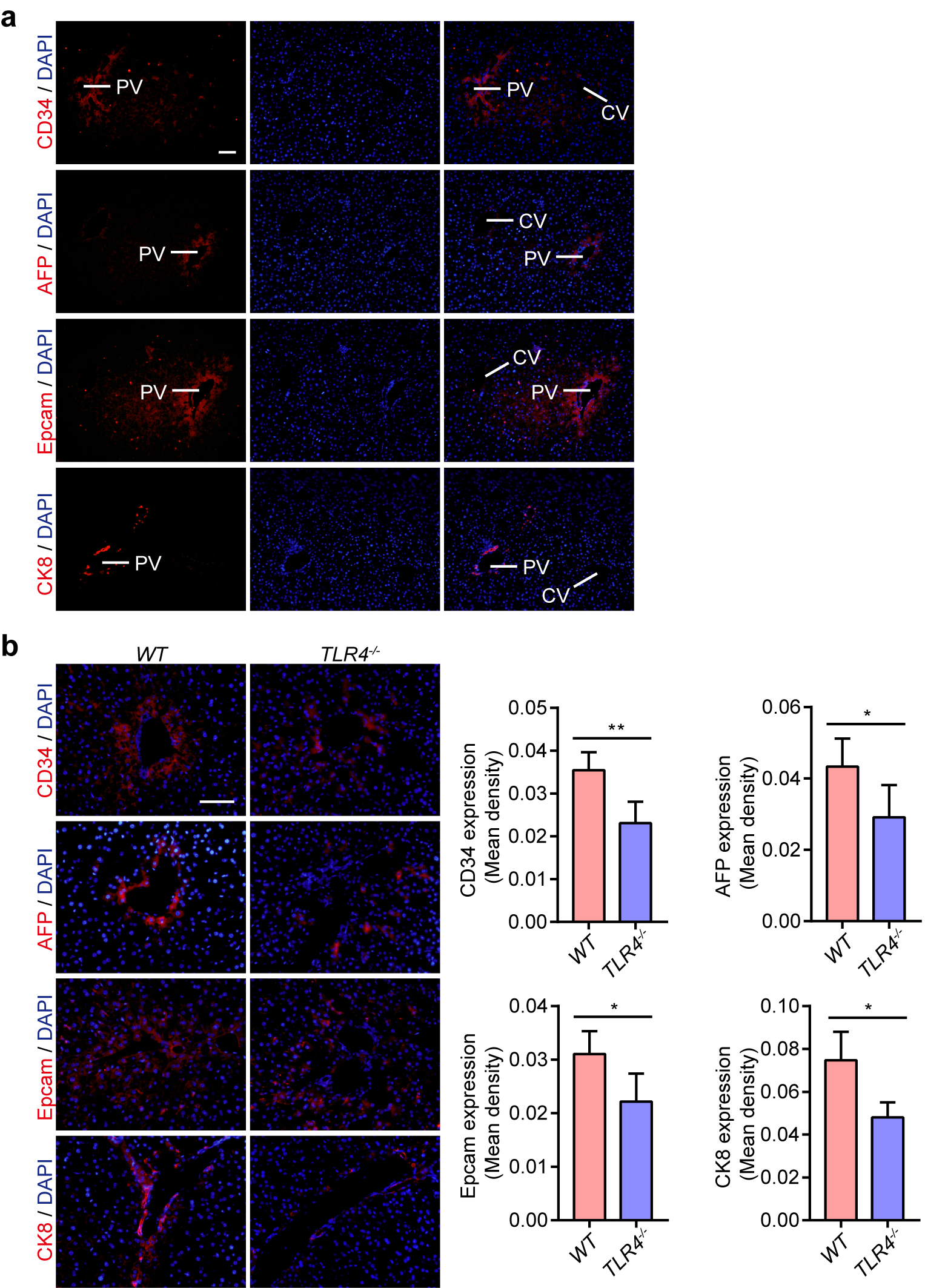

Supplement: Supplementary file 1 — Additional file 1: Supplemental Figure S1. The expression of the hepatic stem cell markers in WT and TLR4-/- mice. a IF staining of the hepatic stem cell markers CD34 (red), AFP (red), Epcam (red), and CK8 (red) in the liver. Nuclei were counterstained with DAPI (blue). Scale bars, 100 μm. b IF staining of the hepatic stem cell markers CD34, AFP, Epcam, and CK8 in the liver of WT and TLR4-/- mice. Mean density was used to evaluate the expression of these markers. Mean density was calculated as follows: Mean density = (IOD Sum) / (Area Sum), where IOD represents integrated optical density. Scale bars, 50 μm. [file 13287_2021_2421_MOESM1_ESM.tif]

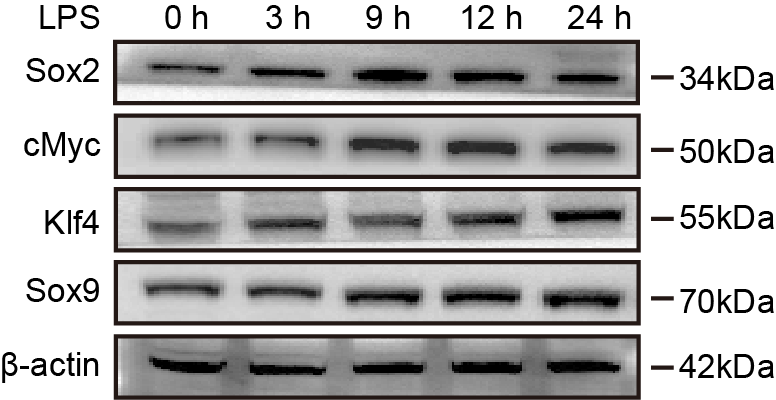

Supplement: Supplementary file 2 — Additional file 2: Supplemental Figure S2. Western blot assay of Sox2, cMyc, Klf4, and Sox9 in primary hepatocytes cultured in basic medium containing LPS for the indicated amounts of time. [file 13287_2021_2421_MOESM2_ESM.tif]

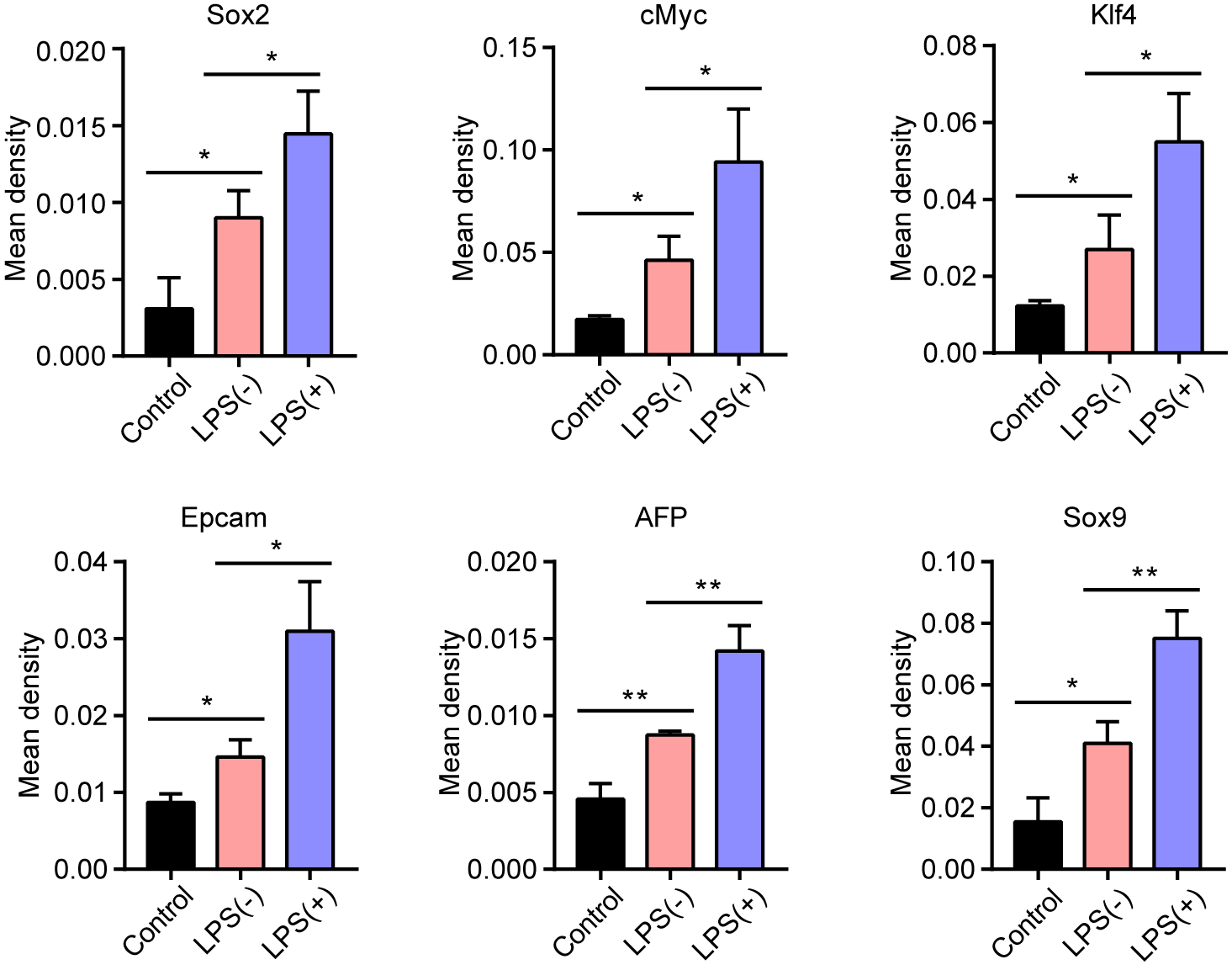

Supplement: Supplementary file 3 — Additional file 3: Supplemental Figure S3. The expression of Sox2, cMyc, Klf4, Epcam, AFP, and Sox9 was measured by mean density. [file 13287_2021_2421_MOESM3_ESM.tif]

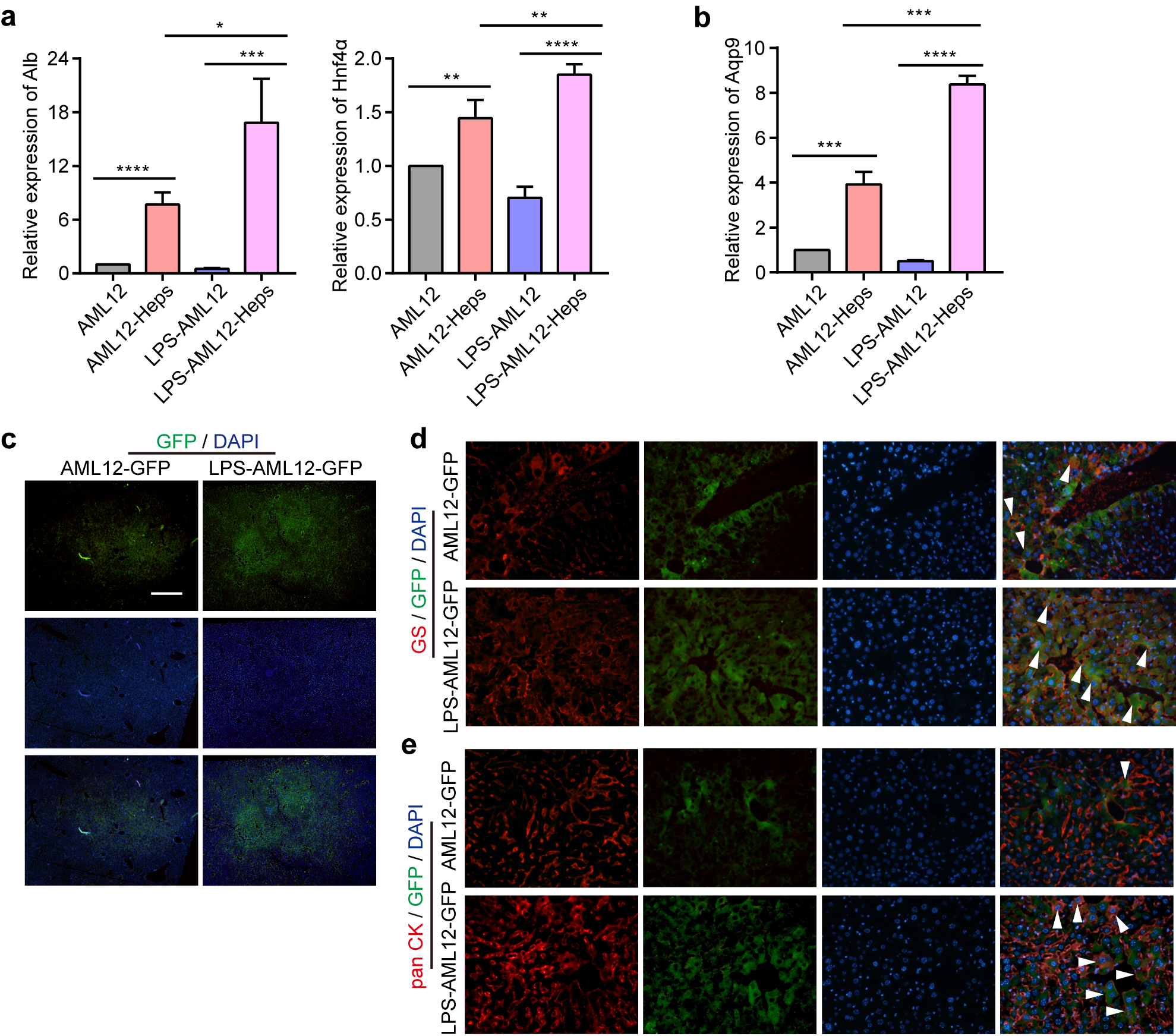

Supplement: Supplementary file 4 — Additional file 4: Supplemental Figure S4. The relative expression of hepatic or biliary markers in LPS-AML12 cells after hepatic or biliary induction. a The relative expression of the mature hepatic marker Alb and Hnf4α in AML12 cells, AML12-Heps, LPS-AML12 cells, and LPS-AML12-Heps as determined by qRT-PCR. b qRT-PCR analysis of the cholangiocyte marker Aqp9 in AML12 cells, AML12-Chols, LPS-AML12, and LPS-AML12-Chols. c Colonization of GFP-tagged AML12 or LPS-AML12 cells in Fah-/- mice 23 days after transplantation. Scale bars, 500 µm. d IF staining of the mature hepatocyte markers GS (red). The arrowheads denote AML12-GFP cells and LPS-AML12-GFP cells with GS staining. Scale bars, 50 µm. e IF staining of live chimaeric Fah-/- mice for the cholangiocyte markers pan CK (red). The arrowheads denote AML12-GFP cells and LPS-AML12-GFP cells with pan CK staining. LPS-AML12: AML12 cells were cultured in reprogramming medium in the presence of LPS for 2 weeks. Scale bars, 50 µm. *P < 0.05, **P < 0.01, ***P < 0.001, ****P < 0.0001. [file 13287_2021_2421_MOESM4_ESM.tif]

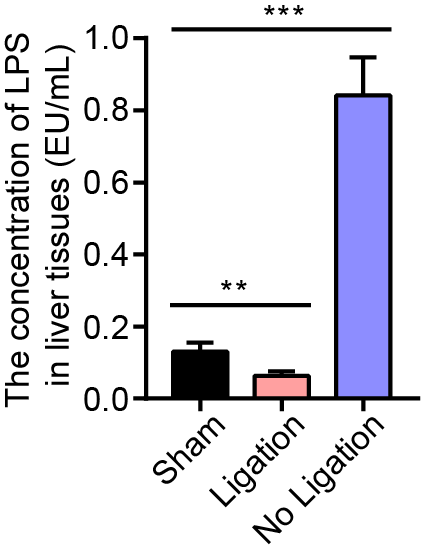

Supplement: Supplementary file 5 — Additional file 5: Supplemental Figure S5. The concentrations of LPS in the different liver lobes from WT mice. **P < 0.01, ***P < 0.001. [file 13287_2021_2421_MOESM5_ESM.tif]
