# Supplementary material for: The stemness of hepatocytes is maintained by high levels of lipopolysaccharide via YAP1 activation
Source: Stem Cell Res Ther. 2021 Jun 10;12:342. doi: 10.1186/s13287-021-02421-7 (PMC8193885; doi:10.1186/s13287-021-02421-7)
Supplement: Supplementary file 6 — Additional file 6: Supplemental Table S1. siRNA target sequences. [file 13287_2021_2421_MOESM6_ESM.docx]

**Supplemental Table S1: siRNA target sequences**

| targets | Sequence 5' to 3' |
| --- | --- |
| siRNA control | TTCTCCGAACGTGTCACGTTT |
| siTLR4 #1 | CAATCTGACGAACCTAGTA |
| siTLR4 #2 | CCATGAACTGACTCTAAGA |
|  |  |
| pDKD-CMV-eGFP-U6-shRNA NC | TTCTCCGAACGTGTCACGT |
| pDKD-CMV-eGFP-U6-shRNA YAP1 | AAGCGCTGAGTTCCGAAATCT |
