# Supplementary material for: The stemness of hepatocytes is maintained by high levels of lipopolysaccharide via YAP1 activation
Source: Stem Cell Res Ther. 2021 Jun 10;12:342. doi: 10.1186/s13287-021-02421-7 (PMC8193885; doi:10.1186/s13287-021-02421-7)
Supplement: Supplementary file 7 — Additional file 7: Supplemental Table S2. Real-time PCR primers. [file 13287_2021_2421_MOESM7_ESM.docx]

**Supplemental Table S2: Real-time PCR primers**

| Primers Names | Sequence 5' to 3' |
| --- | --- |
| Nanog F | CAGAAAAACCAGTGGTTGAAGACTAG |
| Nanog R | GCAATGGATGCTGGGATACTC |
| Sox2 F | GGTTACCTCTTCCTCCCACTCCAG |
| Sox2 R | TCACATGTGCGACAGGGGCAG |
| Oct4 F | TGAGAACCTTCAGGAGATATGCAA |
| Oct4 R | CTCAATGCTAGTTCGCTTTCTCTTC |
| cMyc F | GGAACGAGCTAAAACGGAGCT |
| cMyc R | GGCCTTTTCATTGTTTTCCAACT |
| Klf4 F | AGGAACTCTCTCACATGAAGCG |
| Klf4 R | GGTCGTTGAACTCCTCGGTC |
| Fgf5 F | GAAGCGTCTCACTCCCGAAG |
| Fgf5 R | GAAGAAAACGTCGCGCTACT |
| CD34 F | AAGGCTGGGTGAAGACCCTTA |
| CD34 R | TGAATGGCCGTTTCTGGAAGT |
| CD45 F | ACCACCAGGTGAATGTCAATTT |
| CD45 R | CTTGCTTTCCCTCGGTTCTTT |
| CD90 F | AACACCAACTTGCCCATCC |
| CD90 R | TGTAGTCGCCCTCATCCTT |
| Tert F | TCTACCGCACTTTGGTTGCC |
| Tert R | CAGCACGTTTCTCTCGTTGC |
| Sox9 F | AGTACCCGCATCTGCACAAC |
| Sox9 R | CGAAGGGTCTCTTCTCGCT |
| CK19 F | GGGGGTTCAGTACGCATTGG |
| CK19 R | GAGGACGAGGTCACGAAGC |
| Cyp1a2 F | AGTACATCTCCTTAGCCCCAG |
| Cyp1a2 R | GGTCCGGGTGGATTCTTCAG |
| Ttr F | TTGCCTCGCTGGACTGGTA |
| Ttr R | TTACAGCCACGTCTACAGCAG |
| Aqp1 F | AGGCTTCAATTACCCACTGGA |
| Aqp1 R | GTGAGCACCGCTGATGTGA |
| Alb F | TGCTTTTTCCAGGGGTGTGTT |
| Alb R | TTACTTCCTGCACTAATTTGGCA |
| Hnf4α F | CACGCGGAGGTCAAGCTAC |
| Hnf4α R | CCCAGAGATGGGAGAGGTGAT |
| Aqp9 F | TGGTGTCTACCATGTTCCTCC |
| Aqp9 R | AACCAGAGTTGAGTCCGAGAG |
| β-actin F | GTGACGTTGACATCCGTAAAGA |
| β-actin R | GCCGGACTCATCGTACTCC |
